# Stevens-Johnson Syndrome in Adult Patient Secondary to COVID-19 Infection: Case Report

**DOI:** 10.2196/45062

**Published:** 2023-06-16

**Authors:** Pandharinath Khade, Avani Shah, Vidya Kharkar

**Affiliations:** 1 Department of Dermatology, Venereology, and Leprosy King Edward Memorial Hospital and Seth Gordhandas Sunderdas Medical College Mumbai India

**Keywords:** COVID-19 dermatology, SJS/toxic epidermal necrolysis, infection-induced SJS, infection, rash, Steven-Johnson syndrome, case report, adult patient, skin, skin rash, epidermal necrolysis, male, older adult, skin reaction, allergic reaction, allergy, allergies, toxic epidermal necrolysis, vasculitis, cutaneous, cytokine storm, sequelae, COVID-19, macule, dermatology

## Abstract

COVID-19 is a global pandemic caused by a novel zoonotic RNA virus named SARS-CoV-2. Various cutaneous manifestations associated with COVID-19 have been described, including urticarial rash, confluent erythematous rash, papulovesicular exanthem, chilblain-like acral pattern, livedo reticularis, and purpuric vasculitis pattern. Here, we are presenting a case of a 45-year-old male with mucocutaneous features of Stevens-Johnson syndrome.

## Introduction

COVID-19 is an ongoing global pandemic caused by a novel zoonotic RNA virus named SARS-CoV-2 [1]. Though COVID-19 is known for causing respiratory symptoms, cytokine storms, and thromboembolic sequelae, it has also been reported to be associated with extremely polymorphic cutaneous manifestations [2]. A wide range of cutaneous manifestations associated with COVID-19 has been described, like urticarial rash, confluent erythematous/maculopapular/morbilliform rash, papulovesicular exanthem, chilblain-like acral pattern, livedo reticularis, purpuric vasculitic pattern, and toxic epidermal necrolysis (TEN) or Stevens-Johnson syndrome (SJS) [1,3]. SJS is a rare, severe, life-threatening, adverse drug reaction affecting <10% of the skin and mucous membrane. Some reported cases of infection-induced SJS were caused by mycoplasma pneumonia, viruses, bacterial infections such as streptococcus group A, and mycobacterium [4]. Viruses interact with the immune system and can trigger severe cutaneous adverse reactions in several ways [5]. Here, we report a biopsy-confirmed case of SJS in an adult patient secondary to COVID-19 infection with an unvaccinated status.

## Case Report

**Figure 1:**
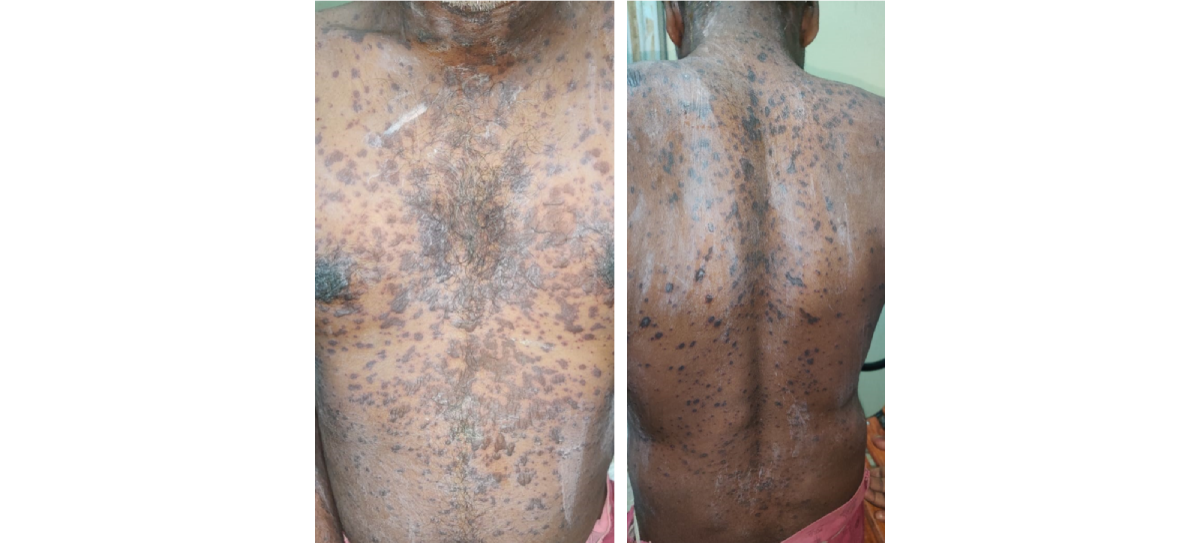
Multiple superficial flaccid blister and violaceous macules on trunk.

**Figure 2:**
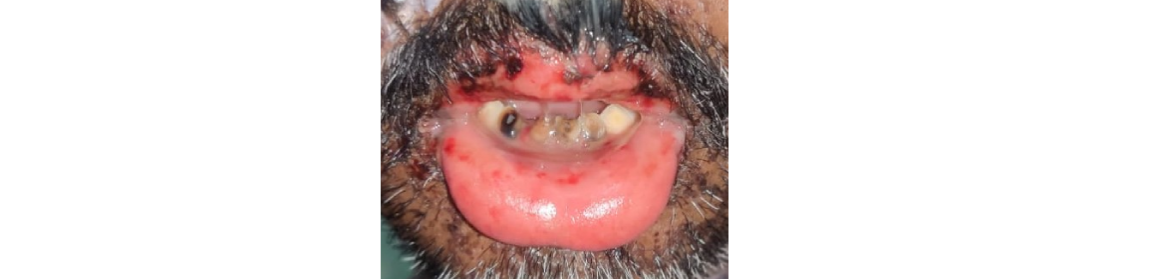
Multiple superficial ulcers and swollen lips.

**Figure 3:**
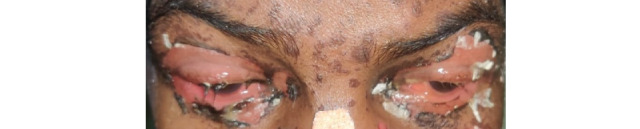
Multiple superficial ulcers involving bilateral upper and lower eyelid with matting of eyelashes.

**Figure 4:**
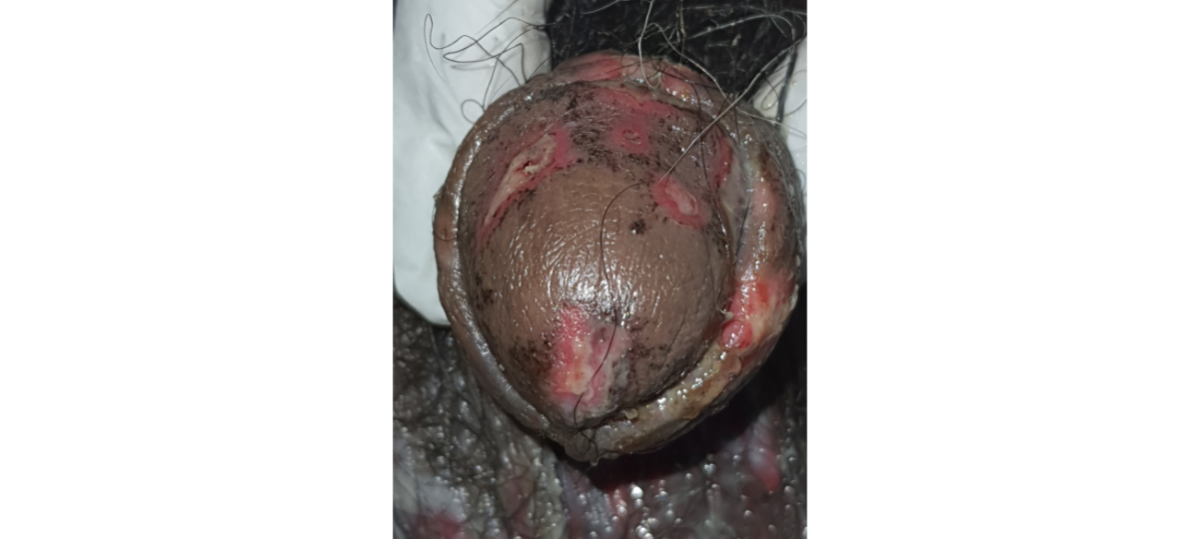
Multiple erosion on glans penis.

A 45-year-old male presented to us with multiple fluid-filled lesions on the upper and lower extremities and raw areas in the oral cavity for 3 days. The patient complained of fever, malaise, and burning of eyes prior to the onset of lesions. The patient denied any history of taking any oral or topical over-the-counter products before the onset of lesions. However, there was an associated history of hypertension and diabetes for which he was taking regular medications for the last 4 years (with no change in medication). The general physical examination was poor. The patient was afebrile, the pulse rate was 130 beats per minute, the SpO_2_ was 96%, and the respiratory rate was 20 cycles per minute. Dermatological examination revealed multiple tender erythematous to purple macules and a few flaccid blisters over the trunk, extremities, and palms and soles (Figure 1). The Pseudo Nikolsky sign was positive. Multiple superficial ulcers were observed on the tongue, lips, eyes, scrotum, and shaft of the penis including the glans penis, with matted eyelashes (Figures 2-4). The systemic examination was unremarkable.

**Figure 5:**
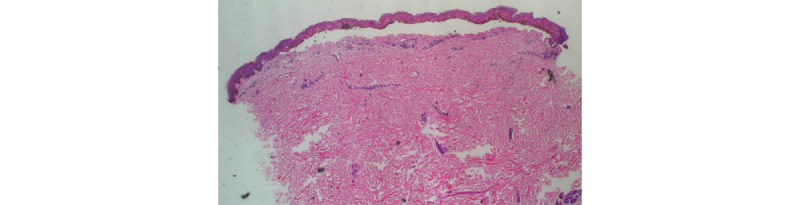
Subepidermal split, spongiosis, necrosis of whole epidermis, and mild superficial perivascular infiltrate (4x).

**Figure 6:**
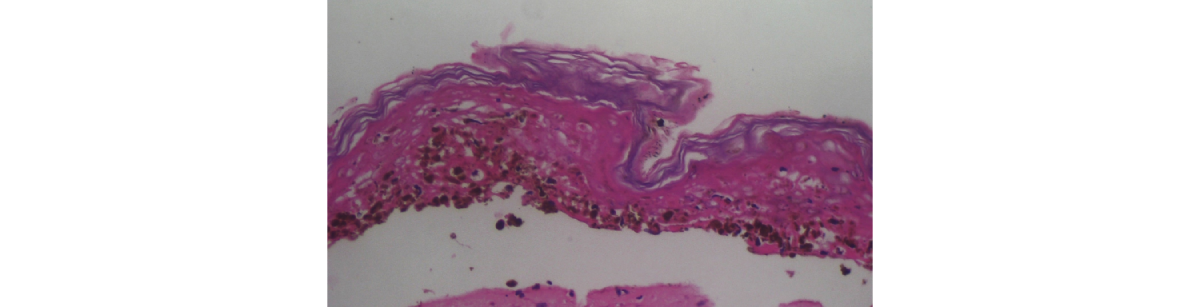
Sheet of epidermal necrosis (40x).

**Figure 7:**
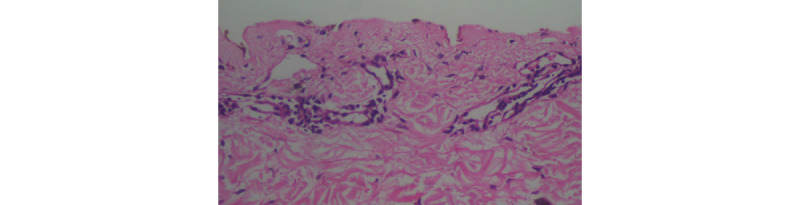
Sparse superficial perivascular lymphocytic infiltrate (40x).

The hematological investigations were normal (hemoglobin: 13 g/dl; white blood cell: 5100 cell/mm^3^; platelets: 1 lac/mcl). Liver function tests, chest x-ray, electrocardiogram, and ultrasonography of the abdomen and pelvis were normal. The patient tested negative for HIV, hepatitis B surface antigen (HBsAg), hepatitis C virus (HCV) antigen, and herpes simplex virus (HSV) IgM and IgG antibodies. However, real-time polymerase chain reaction (RT-PCR) was positive for COVID-19 infection with an elevated C-reactive protein (80.55 mg/L) and erythrocyte sedimentation rate (40 mm/hr). D dimer and lactate dehydrogenase were within normal limits. Histopathological examination of the purple macule showed spongiosis, necrosis of the epidermis, and mild superficial perivascular lymphocytic infiltrate (Figures 5-7). Based on history, clinical examination, and investigations, we confirmed our diagnosis as SJS most likely due to the COVID-19 virus. We informed the patient about his condition and general measures were taken care of: strict isolation and monitoring of temperature, pulse, respiratory rate, blood sugar levels, and urine output were carried out periodically. Fluids and parenteral nutrition were provided intravenously. Injection of 8 mg of dexamethasone thrice daily was started with rapid tapering every 3 days. The patient reported improvement in a span of 10 days.

## Discussion

SJS is a serious life-threatening disease of the skin and mucous membranes [6]. Most cases of SJS/TEN are triggered by drugs, mainly sulfonamides, beta-lactam antibiotics, nonsteroidal anti-inflammatory drugs, and allopurinol. It usually occurs 4-28 days after drug exposure [2,4]. Hence, obtaining a detailed drug exposure history is important. Various microbes, especially viruses, play an active role in triggering an immune response, which leads to SJS/TEN [5]. There have been case reports of SJS/TEN associated with coxsackievirus, influenza virus, Epstein-Barr virus, human herpes virus 6 and 7, cytomegalovirus, and parvovirus infection [4].

However, the exact pathogenesis of infection-induced SJS is unknown, but the immunological response to infectious agents causing generalized apoptosis of keratinocytes by T lymphocytes and proteins like granulysin and Fas ligand has been postulated [7]. The entry of the virus activates the host immune response mechanism. Viral reactivation activates the resident memory T-cells. Resident memory T cells are important cells in infection-induced SJS/TEN, which decide viral control, viral latency, or viral lethality and tissue damage. They release various cytokines like interferon-ɣ, which causes viral clearance and keratinocyte damage [8].

SJS occurrence in patients with COVID-19 has been reported to be associated mostly with medications like paracetamol, naproxen, azithromycin, hydroxychloroquine, allopurinol, cotrimoxazole, lenalidomide, and lamotrigine [4,6,9]. To date, only 3 cases of COVID-19–induced SJS have been reported [10,11].

In this case, our patient was on antihypertensive and antidiabetic medications for 4 years with no change or addition of any other medication. Hence, the possibility of drug-induced SJS was ruled out. In contrast to drug-induced SJS, infection-induced SJS shows more mucosal involvement than cutaneous involvement. This finding is similar to our case [2,12].

As per a study done by Wetter and Camilleri [13], individual necrotic keratinocytes, dense dermal and appendageal infiltrate, red blood cell extravasation, pigment incontinence, parakeratosis, and a substantial number of eosinophils or neutrophils are important features found in drug-related SJS, which were absent in our case [13].

In this case, the patient tested negative for HIV, HBsAg, HCV antigen, and HSV IgM and IgG antibodies and mycoplasma pneumonia antigen. However, our patient’s throat swab was positive for COVID-19 infection (tested by RT-PCR).

Primary COVID-19 infection may have caused the disease through the pathophysiology mentioned above. The immune system can be activated by virus-associated antigen patterns, as well as viral genomes [2,8]. As the course of infection-induced SJS is benign, these patients do not show severe symptoms and show a good response to treatment [12]. We have treated our patient with tapering doses of injection dexamethasone and prophylactic antibiotics as per COVID-19 protocol. Our patient improved in a span of 2 weeks.

Here, we would like to conclude that primary COVID-19 infection may have caused SJS by triggering the immunological response of the host. This causes generalized apoptosis of keratinocytes by T lymphocytes. Therefore, one should suspect COVID-19 infection as a rare etiology of SJS.

## Abbreviations

HBsAg: hepatitis B surface antigen
HCV: hepatitis C virus
HSV: herpes simplex virus
RT-PCR: real-time polymerase chain reaction
SJS: Stevens-Johnson syndrome
TEN: toxic epidermal necrolysis
